# Review of the mite subfamily Arctoseiinae Evans with a key to its genera and description of a new genus and species from Siberia (Parasitiformes, Mesostigmata, Ascidae)

**DOI:** 10.3897/zookeys.233.3862

**Published:** 2012-10-26

**Authors:** Evert E. Lindquist, Olga L. Makarova

**Affiliations:** 1 Invertebrate Biodiversity, Research Branch, Agriculture & Agri-Food Canada, Ottawa, Ontario K1A 0C6, Canada; 2 Severtsov Institute of Ecology and Evolution, Russian Academy of Sciences, Leninski pr. 33, Moscow 119071, Russia

**Keywords:** Gamasid mites, *Maxinia arctomontana*, genus nova, species nova, Arctoseiinae, generic identification key, Arctic, geographical range, *Iphidonopsis* Gwiazdowicz 2004, *Diseius* Lindquist & Evans 1965

## Abstract

We redefine the subfamily Arctoseiinae of the family Ascidae, and describe a new genus, *Maxinia* **gen. n.**, based on a new species, *Maxinia arctomontana* **sp. n.**, whose adults display a combination of attri-butes uniquely different from other genera of the subfamily. The geographical range of *Maxinia arctomontana* is limited by arctic and mountain landscapes of Siberia. This description provides furtherdata on the arctic distribution and morphological diversity of the subfamily Arctoseiinae, which is unusually well represented in that region (26–83% in local gamasid mite faunas). Conceptual problems with the genus *Iphidonopsis* Gwiazdowicz, 2004 are reviewed, and a new combination, *Iphidonopsis magnanalis* (Ma & Yin, 1999) **comb. n.**, is presented for *Iphidozercon magnanalis* Ma & Yin, 1999 from China. The genus *Diseius* Lindquist & Evans, 1965 is provisionally moved from the family Ascidae to the Digamasellidae. A new key to the genera of Arctoseiinae is presented.

## Introduction

The subfamily Arctoseiinae at present numbers ca 90 species (including 19 undescribed species at hand of the genus *Arctoseius* Thor, 1930), among which approximately one-third (30 species, 26 – *Arctoseius*) inhabit Arctic landscapes (Makarova 1999, in press). The separate local faunas within the Arctic include 5–16 arctoseiine species which constitute 26–83 % of the local gamasid mite diversity (Makarova 2012, in press). In this communication we account for a recently discovered, undescribed species with a unique combination of attributes that defies its placement in any of the five, currently recognized genera of Arctoseiinae Evans, 1963. We present a diagnosis of the subfamily, which argues for placement of the new genus in it, a description of the new genus, and detailed descriptions and illustrations of adult female and male of the new species. Previous confusion and disparity concerning the genus *Iphidonopsis* Gwiazdowicz, 2004 is considered. The monobasic genus *Diseius* Lindquist & Evans, 1965, previously allocated by various authors to either the Arctoseiinae or the Ascinae of the family Ascidae, is tentatively moved to the Digamasellidae.

## Material and methods

Setal notation for the idiosoma follows Lindquist and Evans (1965), with some modification (Lindquist 1994). The leg and palpal chaetotaxy complies with Evans (1963, 1964). The poroidotaxy and adenotaxy are given according to Johnston and Moraza 1991, with small modification (Makarova 2003). The length of all shields was measured along the mid-line, and the width at the broadest part (in case of sternal/sternitigenital shield, the width was accepted as the distance between gland openings *gvb*; see Fig. 15). The length of the epigynal shield was measured to include the anterior flap, and the greatest width at the posterolateral corners. The length of all legs and tarsi are given excluding the ambulacrum, and also its pedicel on leg I. The subcapitulum length was measured from its anterior margin excluding projections (internal malae, corniculi). The measurements were carried out on all available specimens and their limits are stated in micrometers (μm). The following ratios were used in the description (Makarova 2000):

lD/wDthe length-to-width ratio of the dorsal shield;


lSt/wSt, lVA/wVAthe length-to-width ratios of the sternal (in females) and ventrianal (in females and males) shields respectively;


lCh/lDthe ratio of the lengths of the movable cheliceral digit and the dorsal shield, %;


lCh/lCothe ratio of the lengths of the movable cheliceral digit and the corniculus.


In the male description, the features common with the female are omitted.

Holotype and most of paratypes are deposited in Zoological Institute, Russian Academy of Sciences (ZIRAS), St.-Petersburg; part of paratype series (6 females and 1 male from Suntar-Khayata Range) in Canadian National Collection of Insects and Arachnids, Ottawa (CNC).

## Taxonomy / Systematics

### Subfamily Arctoseiinae Evans, 1963

The Arctoseiinae was first proposed by Evans (1963) in a rather informal manner, based on the results of his observations on the chaetotaxy of the legs among a wide variety of free-living Gamasina. It was based on a reduced chaetotaxy of the genu and tibia of legs II to IV, and “the nature of the chaetotaxy of the opisthonotal region of the dorsal shield in the female.” A formal and more detailed description of the subfamily, including a key to its genera was presented by Lindquist and Evans (1965). From that description, the subfamily may be based on the following apomorphic characteristics within the family Ascidae:

Dorsal shield of adults entire, with or without lateral incisions; opisthonotal region usually with maximum of four pairs of lateral *S* setae, *S*2 usually not added in change from larva to protonymph. Marginal (*r-R*) series of setae lacking *r*6 in podonotal region and often *R*6 posteriorly; marginal *R* setae on lateral soft cuticle on female, but variably on dorsal shield margin on male. Submarginal (*UR*) setae absent. Female sternal shield with third pair of lyrifissures on its posterior margin; fourth pair of sternal setae on soft cuticle. Sternal setae *st*5 usually on soft cuticle flanking female epigynal shield. Male sternitigenital shield free from, but sometimes abutting ventrianal shield, and often not fully integrated with endopodal strips alongside coxae III–IV. Maximum number of setae on genua I–II–III–IV, respectively, 12-10-8-7; on tibiae, 12-9-7-7; in change from protonymph to deutonymph, seta *pd-*3 not added to genu and tibia I, *pl-*2 not added to genu and tibia II, and *al-*2 not added to genua and tibiae III–IV.

Plesiomorphic attributes of the Arctoseiinae include the following:

Gnathotectum basically bi- or tri-ramous, each process simple or denticulate; cheli- ceral movable digit bidentate in female, unidentate in male, without pointed process on mid-ventral surface, and with fringed arthrodial envelope basally; cheliceral fixed digit with small, setiform pilus dentilis, with usually few teeth restricted to apical half of masticatory surface, and without hyaline serrate rim near base on paraxial surface. Tarsus I usually with a conspicuously lanceolate-tipped seta dorsodistally. In change from protonymph to deutonymph, seta *ad*-3 added to genua I–II and tibia I, *ad*-2 added to tibia II, and *al*-2 added to tibia II.

#### Maxinia gen. n.

urn:lsid:zoobank.org:act:271F12C1-9B82-44F6-BD00-B29F44B05C39

http://species-id.net/wiki/Maxinia

##### Type species.

*Maxinia arctomontana* new species. Monotypic. Genus based on adult female and male material representing one newly described species.

##### Diagnosis.

Adults of *Maxinia* are immediately distinguished from those of other arctoseiinae genera by having a broad, robust dorsal shield; by the female having an apomorphically expansive ventrianal shield that encompasses the metapodal plates anterolaterally and includes setae *JV*1 anteriorly; and by the male having an expansive sternitigenital shield that is fully consolidated with the endopodal strips beside coxae III–IV and abuts an expansive ventrianal shield. In addition, apomorphically, the subcapitulum has a narrow deutosternal groove, and the opisthogaster lacks a setal pair *ZV*1. None of the setae on tarsi II to IV are conspicuously differentiated by thinness, elongation, or curved shape. As in *Arctoseius* Thor, 1930 and *Iphidozercon* Berlese, 1903, genu III carries only seven setae (lacking *pv-*1) on adults; however, adults of those genera have less robust, parallel-sided dorsal shields, retain opisthogastric setae *ZV*1, and their females have free metapodal plates and an anal shield. As in *Zerconopsis* Hull, 1918, adults have robustly sclerotized dorsal and ventral shields. However, adults of *Zerconopsis* have two to several pairs of dorsal shield setae (at least *s*4 and *Z*5) uniquely paddle-like in form, retain opisthogastric setae *ZV*1, eight setae (including *pv-*1) on genu III, and the female ventrianal shield is never expansive enough to include the metapodal plates and setae *JV*1; also, their tarsi II to IV have dorsal proximal setae *ad-*2, *pd-*2 conspicuously elongated and curved.

##### Description.

*Idiosomal dorsum*. Dorsal shield relatively broad, entire, without lateral incisions, well sclerotized; surrounding soft integument smoothly striate; all dorsal shield setae simple, undifferentiated in form; opisthonotal region lacking setae *S*2 (Fig. 9). Dorsal shield with complement of 20 pairs of discernible pore-like structures, of which 4 pairs superficially appear secretory (glandular). Marginal *r-R* series of setae on soft integument in female (Fig. 12), but on dorsal shield edge in male (Fig. 22); *r*-series lacking *r*6, *R*-series with *R*1-6; submarginal *UR*-setae absent. Peritrematal shields uniting with dorsal shield anteriorly and pericoxal strip beside coxa IV posteriorly (Fig. 10). Peritremes somewhat reduced in length.

**Figures 1–2.**
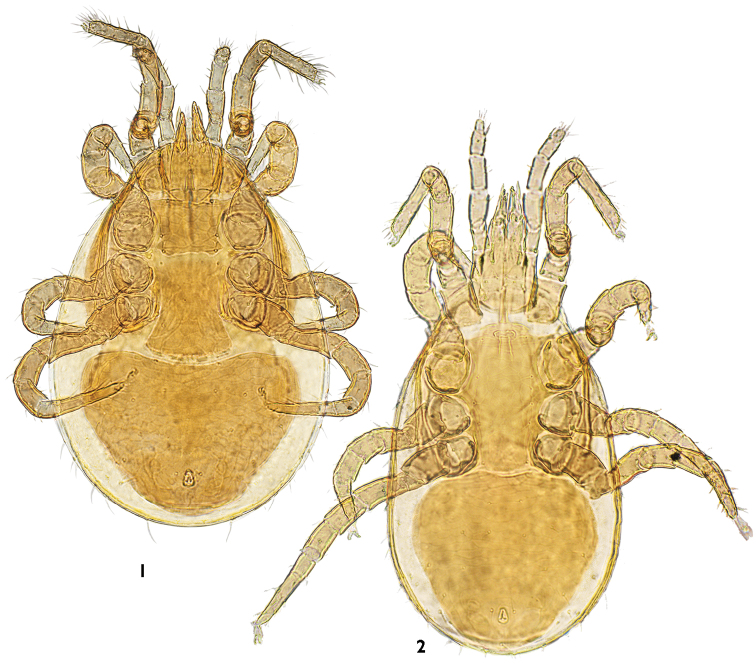
*Maxinia arctomontana* gen. et sp. n., ventral view **1** female **2** male.

*Idiosomal venter*.Tritosternum with laciniae free for most of length (Fig. 17). Ventral shields well sclerotized and ornamented (Figs 1, 2, 12, 22). Female sternal shield entire, continuous with well developed endopodal extensions between coxae I–II, but free from those between coxae II–III (Figs 13–16); endopodal extension between I–II with gland pore apically where approaching or abutting exopodal strip; sternal shield with three pairs of sternal setae and three pairs of poroids; setae *st*4 isolated on soft cuticle. Female with well developed endopodal strips alongside coxae III–IV. Female epigynal shield widened behind level of setae *st*5, its posterior margin broadly convex and nearly abutting ventrianal shield; setae *st*5 and paragenital poroids *iv*5 on soft cuticle; postgenital furrow with two pairs of small platelets. Female with expansive ventrianal shield encompassing metapodal plates anterolaterally and including setae *JV*1 anteriorly, along with other opisthogastric and circumanal setae, except *JV*5, *ZV*4 on soft cuticle; ventrianal shield with paranal setae inserted at level of anterior margin of anus, with gland pores *gv*3 on posterolateral margins, and with cribrum formed as partitioned strip behind level of postanal seta. Male with expansive sternitigenital shield consolidated with presternal platelets and endopodal strips alongside coxae I–IV and abutting expansive ventrianal shield. Male ventrianal shield similar in expansiveness, setation, and other structure to that of female (Fig. 22); setae *JV*5, *ZV*4 on soft cuticle, *ZV*5 absent. Peritrematal shield consolidated with exopodal strips behind coxae IV, with two poroids and one gland pore in area behind stigma, and a gland pore and poroid at level between coxae II–III; exopodal strip alongside coxae II–III usually fragmented.

*Gnathosoma*. Gnathotectum with basically but variably triramous anterior margin (Figs 4–7, 26–28); dorsal face without punctate fields. Chelicerae without any conspicuous process along antiaxial or paraxial lateral surfaces basal to digits (Figs 19, 20, 23); fixed digit with small, setiform pilus dentilis and series of teeth along distal half of masticatory surface; movable cheliceral digit bidentate on female (Fig. 19), unidentate on male (Fig. 23), with arthrodial envelope margin fimbriate; male spermatodactyl simple, digit-like, not recurved basally. Deutosternal groove narrow (Fig. 8), with seven transverse rows of denticles of similar width, rows variably denticulate (2–7 denticles), all rows connected by lateral margins. Corniculi normal in form, parallel in anterior projection from base to apex; internal malae normal in form, similar in length with corniculi or somewhat longer. Subcapitular setae smooth, not greatly disparate in length, *hp*1 not elongated. Palpi with normal setation as described for Gamasina by Evans (1964); palpfemoral seta *al* and palpgenual setae *al*-1 and *al*-2 more or less spatulate distally; palptarsal apotele two-tined.

**Figures 3–10. F2:**
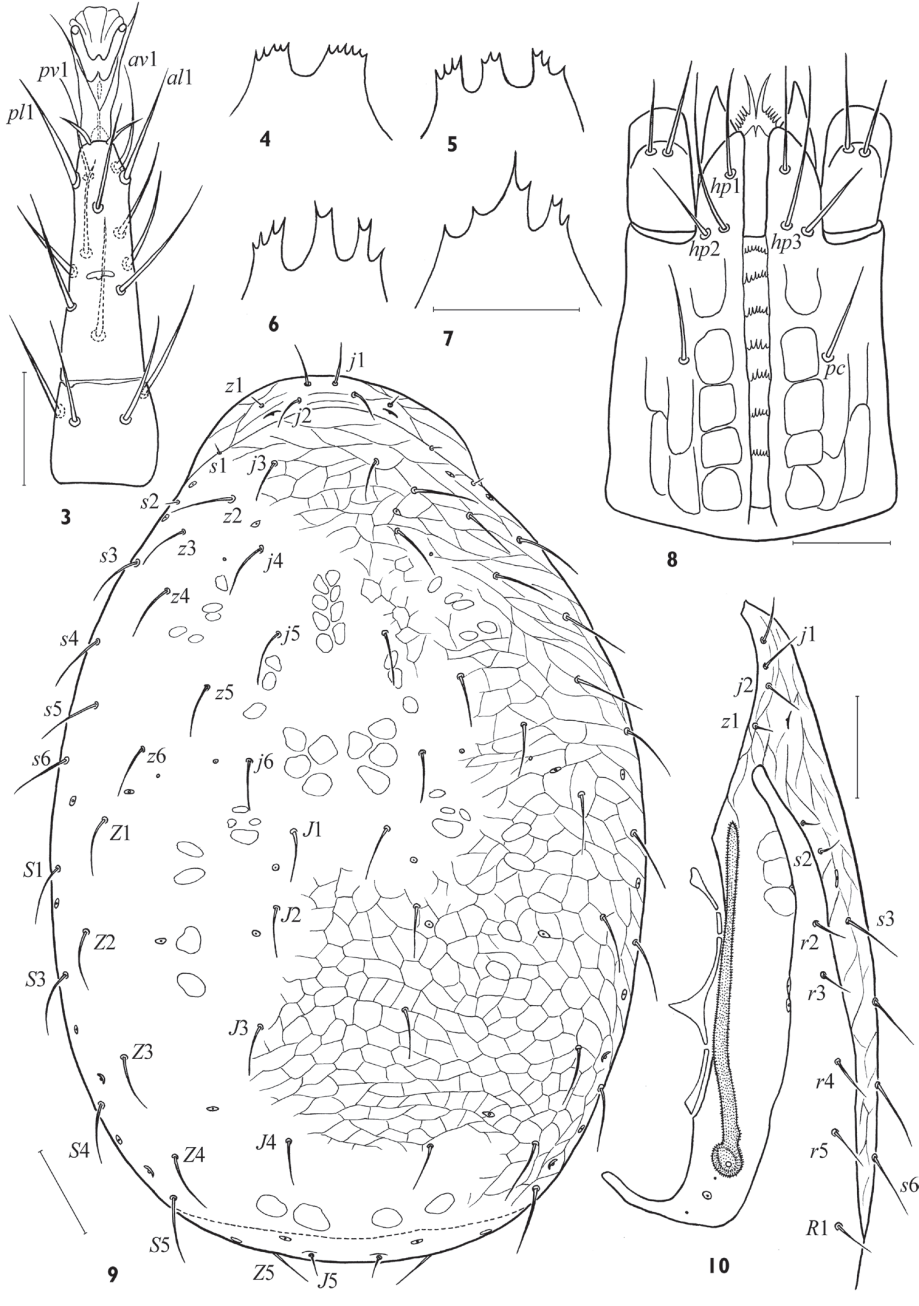
*Maxinia arctomontana* gen. et sp. n., female **3** tarsus II **4–7** gnathotectum **8** subcapitulum **9** dorsal shield **10** peritrematal shield. Scales: **3–8** – 25 μm, **9, 10** – 50 μm.

*Legs*. Legs I to IV with ambulacrum bearing paired claws without basal swelling (Fig. 3), with paradactyli and rounded pulvilli, ambulacrum I smaller than ambulacra II–IV; legs I–IV similar in thickness, and not disparate in length. Legs II–IV with tarsus (excluding ambulacrum) less than twice as long as tibia. Tarsus I without a sensilla distinguishable as *s* by apically lanceolate form (as found in most other members of subfamily), and without markedly elongated setae apically, but with seven finger-shaped sensillae of different lengths. Tarsi II–IV with apical setal processes inconspicuous, shorter than ambulacrum (to base of claws), and with acutely triangular apical process ventrally. Complement of setae on segments of legs I–II–III–IV typical for Arctoseiinae as presented by Lindquist and Evans (1965): femora (2-5/3-2) (2-5/3-1) (1-3/1-1) (1-3/1-1) [Setation of femur III was mistakenly presented as 1-4/1-0 in Lindquist and Evans 1965 (see Evans 1963).]; genua (2-3/2, 2/1-2) (2-3/1, 2/1-1) (1-2/1, 2/0-1) (1-2/1, 2/0-1); tibiae (2-3/2, 2/1-2) (2-2/1, 2/1-1) (1-1/1, 2/1-1) (1-1/1, 2/1-1); in transformation from proto- to deutonymph, seta *pd-*3 not added to genu and tibia I, *pv-*1 but not *pl-*2 added to genu II, and *al-2* not added to genua and tibiae III–IV (this combination of hypotrichy is apomorphic for subfamily). Leg setae collectively smooth; none of setae on tarsi II–IV conspicuously differentiated by thinness, elongation, or curved shape. Legs of male without dimorphically modified setae.

##### Etymology.

The name of the genus is tribute to the first author’s spouse, Maxine Lindquist. Together with him for 55 years, she supported his acarological endeavors, accompanied him in field work throughout North America, and hosted many visiting colleagues at home. The name is feminine in gender.

##### Remarks.

Among some of the characteristics that distinguish them from other taxa of Arctoseiinae, adults of *Maxinia* resemble those of the genus *Neojordensia* Evans, 1957, Ascinae, *e.g*., the relatively broad, well sclerotized dorsal shield, expansive ventrianal shield encompassing the metapodal plates and insertions of setae *JV*1, absence of setae *ZV*1, expansive male sternitigenital shield, and the narrow rows of deutosternal denticles. However, adults of *Neojordensia* present dorsal shield setation and leg chaetotactic attributes typical of the subfamily Ascinae, and are characterized by different distinctive attributes, including absence of paravertical setae *z*1, absences of setae *av-*2 on genu I and *ad-*2 on tibia II, gnathotectum with convex, smooth anterior margin, female with most or all of *r-R* marginal series of setae on margins of dorsal shield, female sternal region with setae *st*1 on separate presternal plates, and female epigynal shield apomorphically widened to include the paragenital poroids as well as setae *st*5 (Lindquist and Evans 1965, Athias-Henriot 1973).

#### Maxinia arctomontana sp. n.

urn:lsid:zoobank.org:act:33101F8A-830E-4F42-958D-FCFB74C3769D

http://species-id.net/wiki/Maxinia_arctomontana

Figs 1 [Fig F2] [Fig F3] [Fig F4] [Fig F5] 35

##### Material.

Holotype, female: EAST SIBERIA, YAKUTIYA, Suntar-Khayata Range, upper reaches of Kyubyume R., 63°13'N, 139°36'E, 1850 m a.s.l., SW slope, bird outlook with gramineous vegetation, 19.VII 2002, O.L. Makarova (ZIRAS).

Paratypes: 16 females, 3 males with same data as for holotype (ZIRAS); 6 females, 1 male, the same district, 1600 m a.s.l., snowbed, litter under *Rhododendron aureum*, 29.VII 2002, O.L. Makarova (CNC); MAGADAN REGION, 8 females, 2 males, 3 protonymphs, 1 larva, Olskoye Plateau, upper reaches of Ola R., 1149 m a.s.l., snowbed, litter under *Rhododendron redowskianum*, 10.VIII 2011, O.L. Makarova (ZIRAS).

Other material: YAKUTIYA, 3 females, vicinity of Ust-Nera settlement, 1400 m a.s.l., litter under *Pinus pumila*, 26.VII 1992, M.B. Potapov; 1 female, Khalerchinskaya Tundra, 69°24'N, 158°37'E, VIII 1991, O.V. Starikova; 4 females, mouth of Kolyma R., 69°32'N, 160°44'E, Pokhodskaya Yedoma, dwarf shrub (*Vaccinium vitis-idaea*, *Betula* sp.) thicket, 18–19.VII 1994, A.B. Babenko; 2 females, delta of Yana R., Shirokostan Peninsula, vicinity of Ledyanoye Lake, *Dryas* community on south slope of valley, 4–6.VIII 1994, A.B. Babenko; 2 females, the same district, date and collector, forb-grassy meadow; MAGADAN REGION, 6 females, 1 male, upper reaches of Kolyma R., Aborigen Mt., *Betula exilis* thicket within icing in valley, litter, 23.VIII 2006, A.A. Alfimov; TAYMYR PENINSULA, 1 female, vicinity of Pyasino Lake, Nyapan’ Upland, *Dryas*-community on hill, 10.VII 1999, O.L. Makarova; 1 female, vicinity of Ragozinka R., 72°57'N, 80°56'E, “lemming hay” on slope, 10.VII 1983, A.B. Babenko; 1 female, mouth of Tareya R., hummocky tundra, 28.VII 2010, O.L. Makarova; 3 females, 3 protonymphs, 72°50'N, 101°15'E, bank of Zakharova Rassokha R., mossy tundra, 3.VII 2011, A.V. Barkalov; YAMAL PENINSULA, 2 females, Seyakha Lake, tundra, litter, 9.VII 1986, V.I. Bulavintsev; 1 female, Yakhadyyakha R., 72°53'N, 70°56'E, lemming hill with *Poa arctica*, 21.VIII 1994, A.B. Babenko; VAIGATCH ISLAND, 1 female, VII 1984, V.I. Bulavintsev; SOUTH SIBERIA, 5 females, Rudnyi Altai Mts., Ivanovskyi Belok Mt., 3.VII 1983, I.P. Vtorov; 1 female, West Sayan Mts., Alashskoye Upland, 2200 m a.s.l., *Dryas*-community, 20.VII 2001, S.K. Stebaeva; 2 females, the same region, vicinity of Sut-Khol Lake, *Larix*-*Vaccinium vitis-idaea*-mosses-community, 27.VII 2001, S.K. Stebaeva.

##### Description.

Middle-sized dark-yellow or brownish mites with rather broad, somewhat pyri-form idiosoma (Figs 1, 2). Idiosomal shields well sclerotized, very finely punctate, with clearly reticulate ornamentation on nearly all surfaces except peritrematal plates. Many setae of body and appendages with fine, hair-like tips, which often are broken off. Dorsal shield rather broad, covering entire dorsal idiosoma, without lateral incisions.

***Female*.** *Idiosomal dorsum*. Dorsal shield 448–520 × 296–356, moderately broad, *lD/wD* ca 1.34–1.62, its maximal width at level of setae *S*1 (Fig. 9). Podonotal region normally with 18 pairs of simple setae (*s*1, *s*2, *z*1 sometimes symmetrically or asymmetrically absent). Opisthonotal region with 14 pairs of setae (*S*2 always absent). Among podonotal setae *z*1, *s*1, *s*2 distinguished by much shorter length (7–11), length of *j*1,2 18–26, other setae 22–36. On opisthonotal region setae *J*1-4 (20–28) shorter than others (30–44), and *J*5 clearly shortest (11–15). Dorsal shield with 4 pairs of cutaneous glands *gdj*4, *gdz*6, *gdZ*3 and *gdZ*4. All marginal setae on soft cuticle (Fig. 12); 4 setae in series *r* (their length 16–24), 6 setae in series *R* (22–38); marginal poroid *Rp* in usual position between setae *R*3 and *R*4.

*Idiosomal venter*. Base of tritosternum narrow (18–22 × 11–14); laciniae with sparse large barbs, free for nearly entire length, fused basal area broadest, with short spicules (Fig. 17); length of lacinia free part 68–78. Presternal platelets 10–16 × 20–24, distinct, lineate, clearly separate from sternal shield (Fig. 12). Sternal shield wider (100–124) than long (60–72), *lSt/wSt* 0.48–0.64, minimal width between coxae II 62–74; consolidated with endopodal platelets between coxae I–II but not between coxae II–III. Endopodal projections between coxae I–II strong, nearly straight, their posterolateral margin concave, apices encompassing opening of gland *gvb*, and abutting or uniting with exopodal extensions (Figs 13–16). Sternal shield entirely reticulated; anterior margin straight or slightly concave; posterior margin straight or slightly concave. Sternal shield with typical setae *st*1-3 (30–34) and lyrifissures *iv*1-3; rarely, vestiges of gland *gv*1 present, off posterior margin of sternal shield. Setae *st*4 (22–28) on soft cuticle. Free endopodal fragments between coxae II–III well sclerotized with angular inner margin. Endopodal strips between coxae III and IV free, well developed (Fig. 18), partly hidden under epigynal flap. Epigynal shield (116–132 × 80–116) distinctly reticulated (Fig. 12), broadly axe- or flask-shaped, with broadly convex hyaline flap not extending to sternal shield, and posterior margin broadly convex; lateral margins strongly widening behind level of setae *st*5, but *st*5 (24–28) and paragenital poroids *iv*5 remain on soft cuticle. Two openings of gland *gv*2 medially to coxa IV and on posterior margin of exocoxal strip behind coxa IV usually poorly visible (Fig. 18). Two pairs of postgenital platelets in fold of soft cuticle. Ventrianal shield expansive, fully reticulated, wider (224–280) than long (165–192), *lVA/wVA* 0.63–0.82, consolidated with metapodal platelet sigillae laterally; anterior margin broadly concave, nearly abutting epigynal shield; posterior margin broadly convex, with cribrum a narrow band subdivided along 8 small festoons. Ventrianal shield with 6 (*JV*1-4, *ZV*2, *ZV*3) opisthogastric setae, plus the circumanal setae; setae similar in moderate length (22–30); paranal setae (20–26) inserted at level of anterior margin of anus, and nearly as long as postanal seta (24–32); opening of gland *gv*3 inconspicuous. Setae *ZV*1 absent, and *ZV*4, *JV*5 on soft cuticle; *JV*5 rather long (32–40). Exopodal strip usually fragmented alongside coxae II–III, mostly contiguous with peritrematal shield but ending freely from it, with extension that abuts or merged with endopodal extensions between coxae I–II (Figs 13–16). Peritrematal shield rather wide (Fig. 10), its anterior end united with dorsal shield, its posterior edge connecting with exopodal platelet enveloping coxa IV posteriorly; lyrifissures *ip*1-3 and glands *gp*1-2present (Fig. 18). Peritreme slightly shortened (152–180 × 9–11), not extending beyond mid-level of coxa I anteriorly. Spermathecal apparatus of laelapoid form, without sclerotized sections (Fig. 21).

**Figures 11–18. F3:**
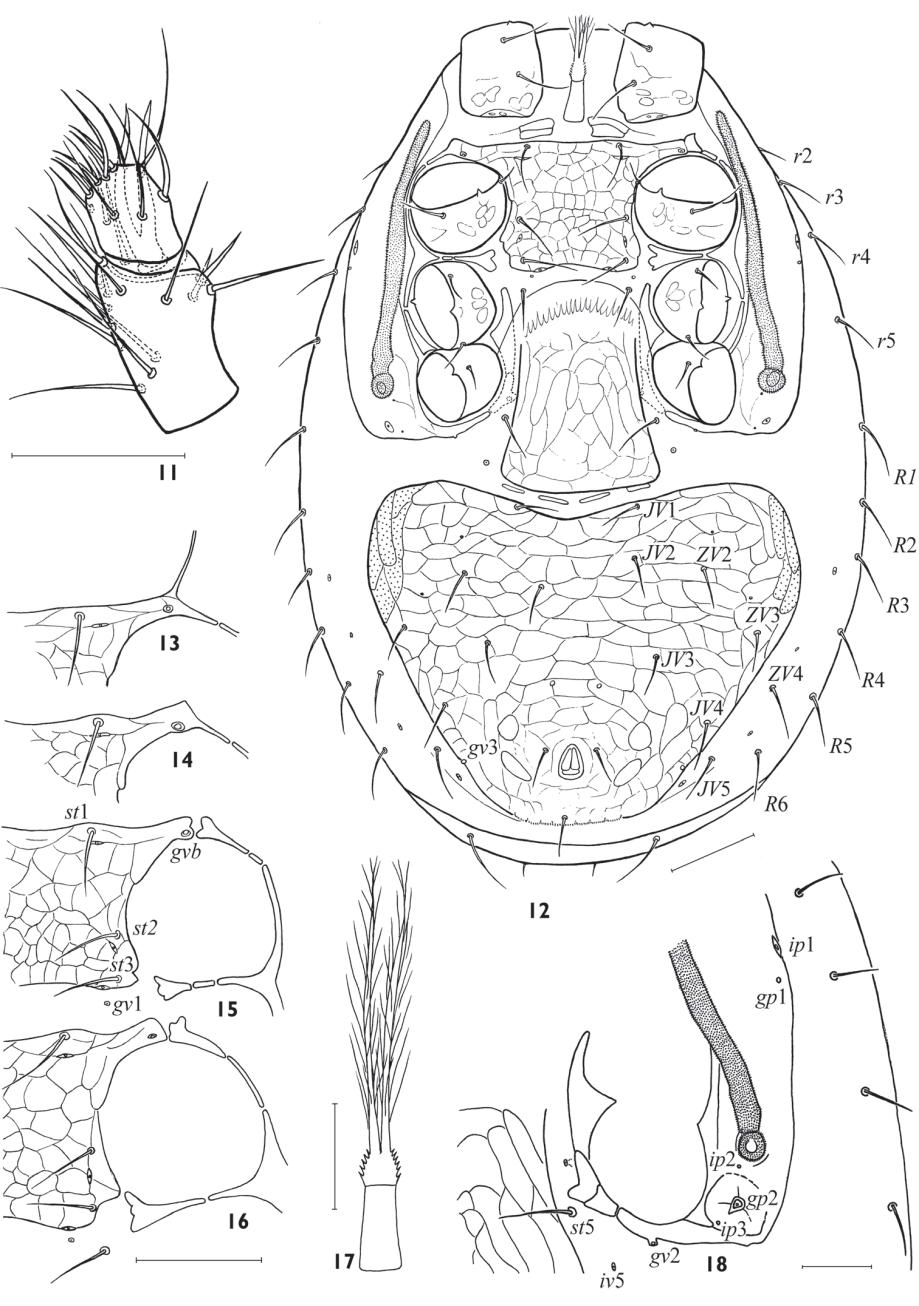
*Maxinia arctomontana* gen. et sp. n., female **11** antaxial view of palpal tarsus and tibia **12** idiosomal venter **13–16** variants of sclerotization of pericoxal region II **17** tritosternum **18** pericoxal region IV. Scales: **11, 17, 18** – 25 μm, **12–16** – 50 μm.

**Figures 19–28. F4:**
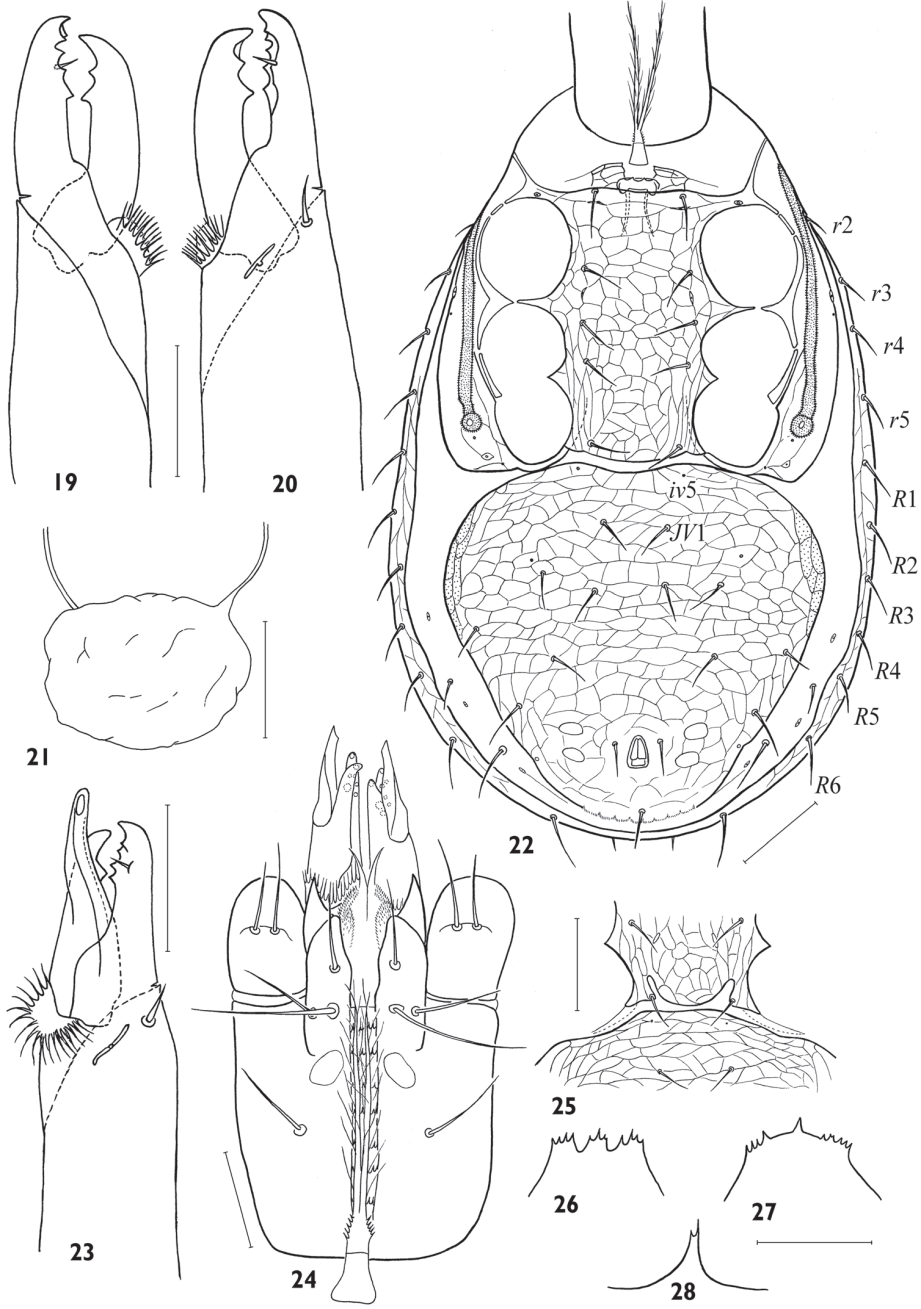
*Maxinia arctomontana* gen. et sp. n., female **(19–21)** and male **(22–28) 19** paraxial view of chelicera **20** antaxial view of chelicera **21** inner part of spermathecal apparatus **22** idiosomal venter **23** antaxial view of chelicera **24** subcapitulum **25** variant of form of posterior margin of sternitigenital shield **26–28** gnathotectum. Scales: **19, 20, 23, 24, 26–28** – 25 μm, **21, 22, 25** – 50 μm.

*Gnathosoma*. Gnathotectum basically triramous, with usually three short, denticulate processes (Figs 4–7), middle projection equal to or longer than lateral ones. Subcapitulum (Fig. 8) longer (98–116) than wide (71–80). Deutosternum with 7 narrow, laterally adjoined rows of denticles (2–7 denticles in each row); groove width 8–9. Hypostomatic pair *hp*3 (39–44) longer than other subcapitular setae (22–30); all setae simple, attenuate. Corniculi of moderate length and width, 30–36 × 11–13. Internal malae slightly longer than corniculi, gradually tapering to tip, with lateral margins fimbriated basally. Chelicera not large (Figs 19–20), its length without basal segment 128–139; cheliceral digits of moderate size (48–57, *lCh/lD* 9.5–12.1 %), one and a half longer than corniculus (*lCh/lCo* 1.48–1.75). Fixed digit of chela ending in apical hook, masticatory surface with one subapical tooth and pilus dentilis in antaxial position and four denticles in paraxial position. Movable digit (44–55) slightly shorter than fixed one, bidentate. Palp length 156–175; internal seta of trochanter (28–32) longer than external seta (19–26); palp with typically specialized setae on femur (*al*) and genu (*al*1, *al*2) thick with oblique tip; palp tarsus without macroseta (Fig. 11).

*Legs*. Legs of moderate length (I 340–396, II 272–304, III 264–296, IV 336–372); leg I shorter than dorsal shield. Length of tarsi I 92–106, II 80–90, III 84–89, IV 102–124. Leg chaetome as described for genus. Setae of legs simple, generally of moderate length; tarsi II–IV each with dorso-proximal setae *ad-*2, *pd-*2 not elongated or curved, and with *al-*1, *pl-*1 not thinner or more elongated than adjacent setae. Ambulacrum I on pedicellate base, claws I (8–10) smaller than claws II–IV (12–15). Tarsus I distally with 7 rod-like solenidia, 5 of them inserted apically. Ambulacra of legs II–IV (length 24–30) with moderately long paradactyli (9–11) extending clearly beyond apices of claws. Tarsi II–IV with apical setae *ad-*1 and *pd-*1 shorter (8–12) than claws (Fig. 3). Four subapical setae on tarsi II–IV evenly distant from apex, almost of equal length, ventral setae *av-*1 and *pv-*1 slightly weaker and shorter (18–23) than lateral setae *al-*1 and *pl-*1 (20–27).

***Male*.** *Idiosomal dorsum*. Dorsal shield 396–424 × 236–276, narrower than in female (*lD/wD* ca 1.50–1.75), fully reticulated, with all *r*-*R* marginal setae (10 pairs) on shield, such that podonotal region with 22 pairs of setae, including *r*2-5, and opisthonotal region with 20 pairs of setae, including *R*1-6 (*S*2 always absent). Relative lengths of setae as on female.

*Idiosomal venter*. Tritosternum base (12–16 × 10–12) shorter than in female (Fig. 22). Presternal platelets connected with sternitigenital shield. Sternitigenital shield fully united with endopodal platelets developed between coxae I–II, coxae II–III, and coxae III–IV, its posterior margin sometimes with pair of indentations (Fig. 25); length of shield 136–158, width between *gvb* openings 92–110, width at midlevel of coxae II 64–72, width at midlevel of coxae IV 60–72. Sternitigenital shield fully reticulated, with setae *st*1-3 (24–30) longer than setae *st*4,5 (20–24), and with lyrifissures *iv*1,2; lyrifissures *iv*3 and vestiges of glands *gv*1 not discernible. Ventrianal shield abutting but free from sternitigenital and peritrematal shields, fully reticulated, expansive, slightly wider (194–224) than long (176–202), yet narrower than in female, *lVA/wVA* 0.82–0.98, consolidated with metapodal platelet sigillae laterally; anterior margin straight or slightly concave, posterior margin and cribrum formed as in female. Ventrianal shield with setation and form, placement, and lengths of setae as in female, except *JV*1 more removed from anterior margin and poroids *iv*5 present on shield. Setae *ZV*1 absent, *ZV*4 and *JV*5 on soft cuticle.

*Gnathosoma*. Apices of gnathotectum similar to female but sometimes variously shaped in different ways (Figs 26–28). Corniculi and internal malae as in female (Fig. 24). Cheliceral digits (38–43, *lCh/lD* 9.1–10.7 %) longer than corniculus (30–32 × 11–12, *lCh/lCo* 1.22–1.34). Fixed digit with dentition similar to female (Fig. 23) except smaller number of denticles in paraxial row (2–3). Movable digit with one large denticle and simple, closed, trough-like spermatodactyl (length of free part 22–24), protruding shortly beyond tip of digit.

*Legs*. Length of legs I–IV 344–376, 272–284, 256–264 and 320–340 respectively; length of tarsi I–IV 96–101, 78–80, 76–80 and 100–112 respectively. Legs without dimorphically modified setae.

***Deutonymph*.** Unknown.

**Figures 29–34. F5:**
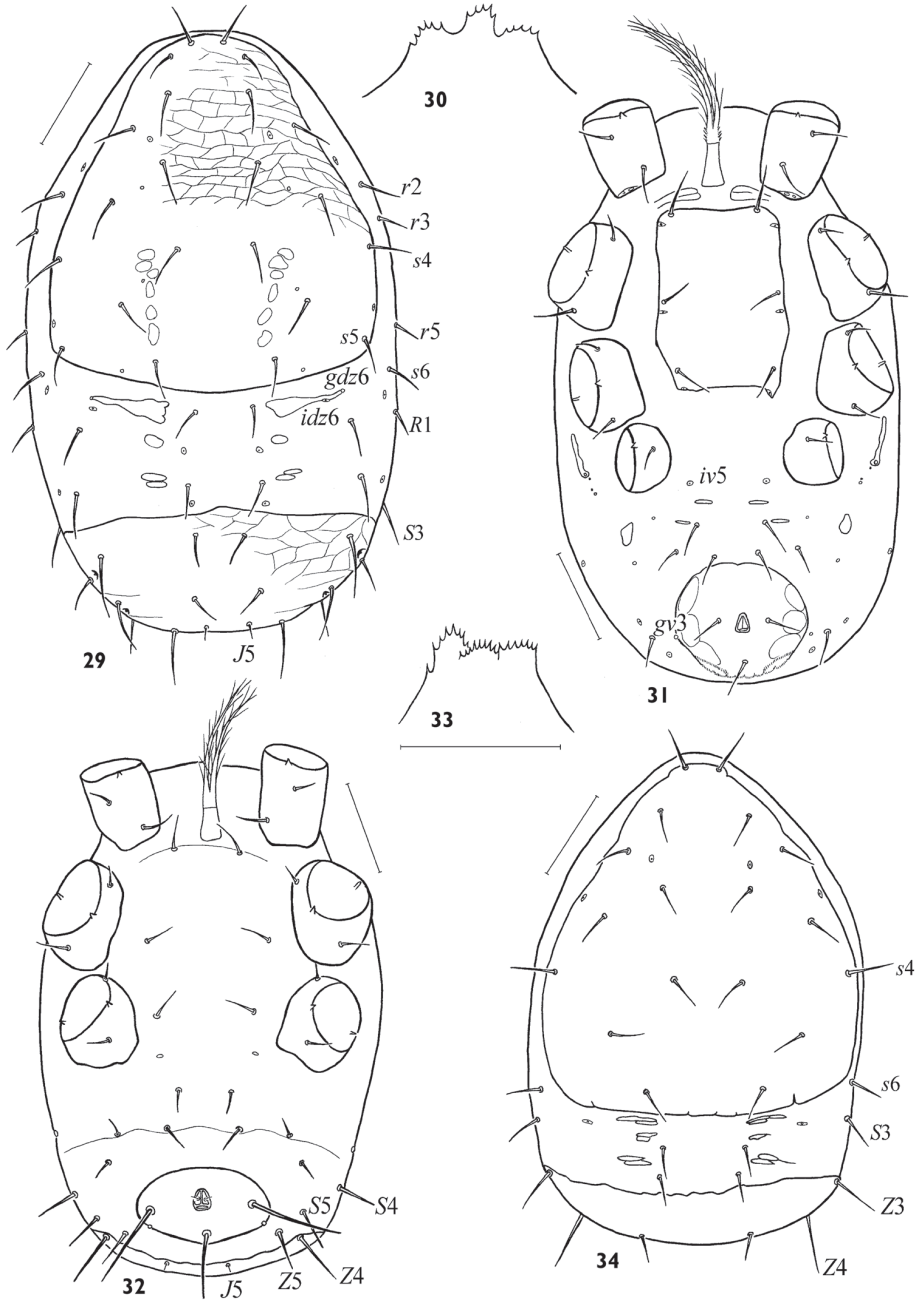
*Maxinia arctomontana* gen. et sp. n., protonymph **(29–31)** and larva **(32–34) 29** idiosomal dorsum **30** gnathotectum **31** idiosomal venter **32** idiosomal venter **33** gnathotectum **34** idiosomal dorsum. Scales: **29, 31, 32, 34** – 50 μm, **30, 33** – 25 μm.

***Protonymph***.* Idiosomal dorsum*. Idiosoma 304–320 × 185–212. Podonotal shield 188–200 × 176–184, reticulate, with 11 pairs of setae (Fig. 29) and no less than 5 pairs of pore-like structures including opening of gland *gdj*4. Pygidial shield 54–76 × 160–164, reticulate, with 8 pairs of setae and openings of glands *gdZ*3, *gdZ*4. Interscutal soft cuticle with 7 pairs of setae and 3 pairs of mesonotal sclerites, anterior sclerites bearing gland pores *gdz*6 and poroids *idz*6. Setae *r*2, *r*3, and *r*5 on lateral soft cuticle beside podonotal shield. All dorsal setae needle-shaped, their length 14–35, except *J*5 clearly shortest (7–8).

*Idiosomal venter*. Tritosternum as in adults (Fig. 31). Sternal shield poorly sclerotized, with 3 pairs of setae (20–24) and 3 pairs of lyrifissures; presternal platelets weakly developed. Seta *st*5 absent, but poroid *iv*5 distinct. Anal shield nearly circular, with paranal setae nearly as long as postanal seta, and usually with opening of gland *gv*3. Opisthogastric region with 2 pairs of fine medial sclerites, small (10–13 × 7–8) metapodal platelets, 4 pairs of setae and 5 pairs of poroids. Peritremes extending to posterior margins of coxae III.

*Gnathosoma*. Gnathosomal structures in general as in adults, tectum as in Fig. 30.

*Legs*. Leg chaetotaxy typical for protonymphs of Ascidae (Lindquist and Evans 1965).

***Larva*.** *Idiosomal dorsum*. Idiosoma 260 × 180. Podonotal and pygidial shields smooth, bearing 9 and 4 pairs of needle-shaped setae, respectively (Fig. 34). Interscutal soft cuticle with 3 pairs of narrow sclerites and 4 pairs of setae. Pygidial shield bent caudally, so setae *J*5 and *Z*4 inserted on ventral surface.

*Idiosomal venter*. Tritosternum in general as in adults (Fig. 32). Intercoxal region without delineated sternal shield, with 3 pairs of setae (17–18), lacking lyrifissures but with typical subdermal pair of structures between bases of legs III. Ventral soft cuticle with transverse fold nearly delineating podosomatic from opisthogastric regions. Opisthogastric soft cuticle with typical 4 pairs of opisthogastric setae plus ventrolaterally displaced dorsal setae *S*4, *S*5, *Z*5. Anal shield (38 × 78), transversally oval, wider than long, with circumanal setae large, paranals (54) longer than postanal (36), and with gland opening *gv*3 nearly posterior to paranal setae; anal valves with tiny euanal setae; cribrum undeveloped.

*Gnathosoma*. Hypostome, gnathotectum (Fig. 33), chelicera similar to adults, setae *hp*-3 and *pc* absent.

*Legs*. Leg chaetotaxy typical for larvae of Ascidae (Lindquist and Evans 1965).

##### Etymology.

The species name indicates its geographical range.

##### Distribution.

**Figure 35.**
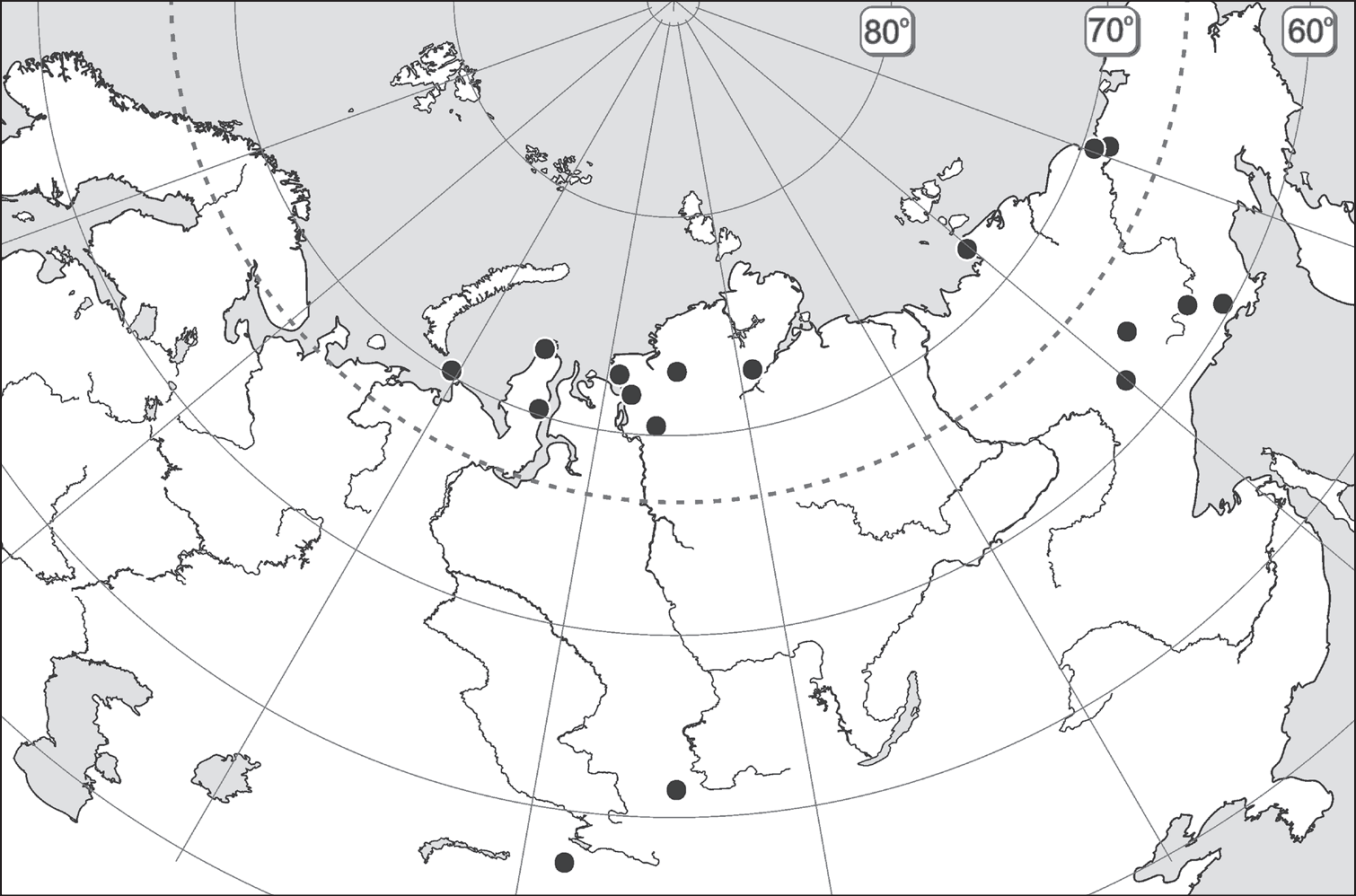
Records of*Maxinia arctomontana* gen. et sp. n.

At present known from zonal and mountainous tundra landscapes of West, Middle and East Siberian sectors (Fig. 35).

##### Ecology.

Recorded from dry and humid tundra sites, meadows including zoogenic ones (e.g. bird of prey outlooks) and shrub (*Pinus pumila*, dwarf *Betula*, *Rhododendron*) communities.

##### Variability.

The species is rather stable morphologically. The variability concerns mainly the form of gnathotectum (Figs 4–7, 26–28), the degree of coalescence of endocoxal and exocoxal elements around coxa II (Figs 13–16), sometimes the symmetrical or asymmetrical absence of setae *s*1, *s*2, *z*1, and the numbers of denticles in the rows on the deutosternum (usually 2–5 or 3–7).

## Discussion

### Notes on two problematic genera of Arctoseiinae

Genus *Iphidonopsis*

In the following key, *Iphidonopsis* Gwiazdowicz, 2004 is treated in the original sense (Gwiazdowicz 2004) to include *Iphidonopsis sculptus* Gwiazdowicz, 2004 and *Iphidonopsis magnanalis* (Ma & Yin, 1999) **comb. n.** Subsequently, Gwiazdowicz (2007) broadened his concept of *Iphidonopsis* to accommodate other species (e.g., *Lasioseius pulvisculus* Berlese, 1921, *Seiulus minutus* Halbert, 1915) and thereby distinguished his genus from *Arctoseius* Thor, 1930, primarily on the dorsal shield of adults having lateral incisions in *Arctoseius*, and these lacking in *Iphidonopsis*. However, not only is the lack of such incisions a plesiomorphic character state, as presented in some other genera of Arctoseiinae, but the presence of lateral incisions varies greatly among species, and is even subject to geographical variability and sexual dimorphism among some species, in *Arctoseius*. So, Athias-Henriot (1959, 1961) reported the lack of lateral incisions on females of *Arctoseius pannonicus* Willmann, 1949 from Corsica and Spain, although shallow ones are present in other parts of Europe. Observations by Lindquist (1964) also indicated these differences between some populations. Illustrations of *Arctoseius venustulus* (Berlese, 1917) by Bernhard (1963) show just the slightest of lateral incisions on females, and entire lateral margins on males. Also, since *Seiulus minutus* has been previously designated as type-species of the genus *Arctoseiopsis* by Evans (1954), Gwiazdowicz’s placement of this species in *Iphidonopsis* would render this genus a junior synonym of *Arctoseiopsis*. With added phylogenetic perspective, we continue to follow the previous concept of Lindquist and Evans (1965), such that the genus *Arctoseius* embraces *Arctoseiopsis* and includes the species *Arctoseiopsis minutus*, *Arctoseiopsis pulvisculus*, and *Arctoseiopsis venustulus*. Adult females of *Iphidonopsis* are somewhat similar to those of *Arctoseius* and *Iphidozercon* in retaining what may be considered to be an apomorphically reduced, deutonymphal form of anal shield. However, as indicated in the following key, the genus *Iphidonopsis* shares with *Zerconopsis* and *Xenoseius* Lindquist & Evans, 1965 the apomorphic attribute of having dorsoproximal setae *ad*-2 and *pd*-2 of tarsi II to IV elongated, curved, somewhat whip-like, while retaining the plesiomorphic attribute of genu III having eight setae, including *pv*-1. In contrast, *Arctoseius*, *Iphidozercon* and *Maxinia* are plesiomorphic in having setae *ad*-2 and *pd*-2 of tarsi II to IV unmodified, but sharing the apomorphic attribute of genu III having seven setae, in absence of *pv-*1.

### Genus *Diseius*

Provisionally, we exclude the monotypic genus *Diseius* Lindquist & Evans, 1965 from the Arctoseiinae and from the following key to its genera. Based exclusively on gnathosomatic and idiosomatic attributes, the type-species of this genus, *Iphidozercon ulmi* Hirschmann, 1962, has been assigned to various genera as conceived by different authors’ concepts of the family Ascidae, i.e., to the genus *Iphidozercon* (including *Arctoseius*, *Leioseius* Berlese, 1916 and *Gamasellodes* Athias-Henriot, 1961) sensu Hirschmann (1962), to the subgenus *Leioseius (Leioseius)* (including *Gamasellodes* but excluding *Arctoseius* and *Iphidozercon*) by Bernhard (1963), and to the genus *Leioseius* (including *Gamasellodes* but excluding *Arctoseius* and *Iphidozercon*) by Karg (1971, 1993). Accounting for those attributes and additional ones of leg chaetotaxy, the species was assigned to a separate, new genus and placed in the tribe Ascini of the subfamily Ascinae (separate from the subfamily Arctoseiinae), by Lindquist and Evans (1965). The chaetotaxy of the genu and tibia of legs III and IV of *Diseius ulmi* resembles that of genera of Arctoseiinae, but *Diseius* was excluded from the Arctoseiinae based primarily on the adult female having a fully divided dorsal shield, and having sternal setae *st*5 inserted on the edges of the epigynal shield, rather than on soft cuticle. However, new observations of female *Diseius* by one of us (EEL) revealed the unanticipated absence of setae *al-*3 and *pl-*3 on basitarsi III–IV, and also of *pd-*3 on basitarsus IV. The chaetotaxy of tarsi II to IV is extremely constant throughout the families of free-living Gamasina (Evans 1963). Losses of basitarsal setae on legs II to IV are not known to occur among species of Ascidae, but they have been noted sporadically in the Digamasellidae and Rhodacaridae (Lindquist 1975). Also, in the absence of *j*2, setae *j*3 are inserted relatively closely behind *j*1 in *Diseius*, much as in the digamasellid subgenus *Longoseius (Longoseius)* Chant, sensu Lindquist (1975). The subcortical habitat association of *Diseius ulmi* with scolytid beetles is in common with that of a variety of digamasellids. As adult females of *Diseius ulmi* are highly neotenous in both leg and dorsal idiosomal setation, the similarity of leg chaetotaxy on genua and tibiae II–IV with Arctoseiinae is thought to be convergent, and our new observations persuade us to place *Diseius* tentatively in the Digamasellidae instead of the Ascidae, until such time as additional attributes become available from description of the female spermathecal system and of attributes of the adult male.

### Key to genera of Arctoseiinae. Adults

**Table:** 

1	Leg chaetotaxy reduced: seta *pd-*3 absent on genu and tibia I, *pl-*2 absent on genu and tibia II, *al-*2 absent on genua and tibiae III-IV (*maximum* number of setae on genua I to IV, 12-10-8-7; on tibiae I-IV, 12-9-7-7); adult dorsal shield entire, with or without lateral incisions; opisthonotal region of dorsal shield usually with 4 pairs of lateral setae (*S*2 usually absent, exception – some *Xenoseius* Lindquist & Evans, 1965)	Arctoseiinae (6 genera)...2
–	Leg chaetotaxy without above reductions: *pd-*3 present on genu and tibia I, *pl-*2 present on genu and tibia II, *al-*2 present on genua and tibiae III-IV (*minimum* number of setae on genua I to IV: 12-11-8-9; on tibiae I-IV: 12-9-8-10); adult dorsal shield entire or divided; opisthonotal region of dorsal shield usually with 5 pairs of lateral setae (*S*2 present)	Ascinae (11 genera)
2	Genu III usually with 8 setae (*pv*-1 present); tarsi II–IV each with dorso-proximal setae *ad*-2, *pd-*2 elongate, curved	3
–	Genu III with 7 setae (*pv*-1 absent); tarsi II–IV with dorso-proximal setae not elongate or curved	5
3	Female with broad anal shield bearing only three circumanal setae; subcapitulum with hypostomatic setae *hp*-1 only slightly elongated, about 1.2 as long as *hp*-3;dorsal shield without midlateral incisions	*Iphidonopsis* Gwiazdowicz, 2004
–	Female with ventrianal shield bearing 1–6 pairs of opisthogastric setae in addition to circumanal setae; subcapitulum with anterior pair of hypostomatic setae *hp*-1 conspicuously elongated, at least 1.5 as long as *hp*-3;dorsal shield with or without midlateral incisions4
4	Tarsus I without pretarsus and claws; setae *j*1 and *z*1 smooth or barbed and variable in length; all other dorsal setae simple, none paddle-shaped	*Xenoseius* Lindquist & Evans, 1965
–	Tarsus I with pretarsus and claws; setae *j*1 and *z*1 smooth, pointed, or *j*1 rarely paddle-shaped; some dorsal setae (at least *s*4 and *Z*5) always paddle-shaped	*Zerconopsis* Hull, 1918
5	Tarsi II–IV with 1 (*al-*1) or 2 (*al*-1, *pl*-1) dorso-lateral subapical setae very slender and elongate; vertex of dorsal shield strongly arched downward, setae *j*1 concealed from above; peritremes sharply recurved anteriorly; dorsal shield without midlateral incisions; palptarsus with macroseta	*Iphidozercon* Berlese, 1903
–	Tarsi II–IV with neither of dorso-lateral subapical setae slender and elongate; vertex of dorsal shield not strongly arched downward, setae *j*1 visible from above; peritremes not recurved anteriorly; dorsal shield with or without midlateral incisions; palptarsus without macroseta	6
6	Dorsal shield of adult with parallel sides, lightly to moderately sclerotized, with lateral incisions of deutonymph retained or obliterated on adult, in male sometimes bearing some marginal setae of series *r* but none of *R* (these free on soft cuticle in female); opisthogastric setae *ZV*1 present; female with anal shield distant from metapodal plates and setae *JV*1; male with sternitigenital shield incompletely consolidated with endopodal strips alongside coxae I–IV and abutting or removed from variably expansive ventral/ventrianal shielding	*Arctoseius* Thor, 1930
–	Dorsal shield of adult relatively broad, well sclerotized, entire, in male bearing all marginal *r*-*R* setae (these free on soft cuticle in female); opisthogastric setae *ZV*1 absent; both sexes with expansive ventrianal shield encompassing metapodal plates anterolaterally and including setae *JV*1 anteriorly; male with expansive sternitigenital shield fully consolidated with endopodal strips alongside coxae I–IV and abutting expansive ventrianal shield	*Maxinia* gen. n.

## Supplementary Material

XML Treatment for Maxinia

XML Treatment for Maxinia arctomontana
